# Pathophysiology, clinical manifestation, and treatment of tuberculosis-associated chronic obstructive pulmonary disease: a narrative review

**DOI:** 10.12771/emj.2025.00059

**Published:** 2025-03-19

**Authors:** Joon Young Choi

**Affiliations:** Division of Pulmonary and Critical Care Medicine, Department of Internal Medicine, Incheon St. Mary’s Hospital, College of Medicine, The Catholic University of Korea, Seoul, Korea

**Keywords:** Bronchodilator agents, Chronic obstructive pulmonary disease, Muscarinic antagonist, Smoking, Tuberculosis

## Abstract

Chronic obstructive pulmonary disease (COPD) is a leading cause of respiratory morbidity and mortality, most often linked to smoking. However, growing evidence indicates that previous tuberculosis (TB) infection is also a critical risk factor for COPD. This review aimed at providing a comprehensive perspective on TB-COPD, covering its epidemiologic significance, pathogenesis, clinical characteristics, and current management approaches. Tuberculosis-associated chronic obstructive pulmonary disease (TB-COPD) is characterized by persistent inflammatory responses, altered immune pathways, and extensive structural lung damage—manifested as cavitation, fibrosis, and airway remodeling. Multiple epidemiologic studies have shown that individuals with a history of TB have a significantly higher likelihood of developing COPD and experiencing worse outcomes, such as increased breathlessness and frequent exacerbations. Key pathogenic mechanisms include elevated matrix metalloproteinase activity and excessive neutrophil-driven inflammation, which lead to alveolar destruction, fibrotic scarring, and the development of bronchiectasis. Treatment generally follows current COPD guidelines, advocating the use of long-acting bronchodilators and the selective application of inhaled corticosteroids. Studies have demonstrated that indacaterol significantly improves lung function and respiratory symptoms, while long-acting muscarinic antagonists have shown survival benefits.

## Introduction

### Background

Chronic obstructive pulmonary disease (COPD) is a major global health concern characterized by persistent respiratory symptoms and irreversible airflow limitation, resulting in a substantial socioeconomic burden [1,2]. COPD has long been attributed to prolonged exposure to harmful particles and gases, most notably from cigarette smoke [3]. However, studies indicate that additional risk factors—including genetic predispositions, early life disadvantages, infections, air pollution, and occupational hazards—may also contribute to its development [4,5]. An analysis of the Korean COPD Subgroup Study cohort found that 39% of COPD patients had a history of pulmonary infection (referred to as COPD-I) [6].

Tuberculosis (TB) remains one of the world’s deadliest infectious diseases, affecting approximately 10 million people annually despite significant advances in diagnosis and treatment [7]. Although improvements in TB treatment have markedly reduced mortality, the long-term pulmonary sequelae of TB are increasingly recognized as a major cause of chronic respiratory impairment, including airflow limitation, bronchiectasis, and fibrosis [8]. These changes may lead to persistent respiratory symptoms and a reduced quality of life [8]. Previous studies have demonstrated that individuals with a history of TB face a higher risk of developing COPD compared to the general population [9-14]. The overlap between post-TB lung disease and COPD may complicate diagnosis and give rise to unique clinical manifestations [15].

### Objectives

This article aims to review current knowledge on the epidemiology, pathogenesis, clinical presentations, and therapeutic strategies related to TB-associated COPD (TB-COPD).

## Ethics statement

As this study is a literature review, it did not require institutional review board approval or individual consent.

## Epidemiology and global burden

The global impact of both TB and COPD is profound, significantly contributing to morbidity and mortality [3]. Despite remarkable advances in TB diagnosis and treatment, TB remains a leading infectious disease, with the World Health Organization reporting approximately 10 million new cases annually [7]. High-burden regions such as Southeast Asia, Africa, and the Western Pacific continue to experience a high prevalence of TB [16]. Moreover, individuals with low socioeconomic status and older adults are at higher risk for TB [17-19]. Importantly, a substantial number of TB survivors develop long-term pulmonary sequelae, which are increasingly recognized as a major contributor to the burden of chronic respiratory diseases [8].

COPD, traditionally regarded as a disease associated with cigarette smoking, is now understood to have a multifactorial etiology [20]. Previous studies have demonstrated that a prior TB infection is a significant risk factor for developing COPD. In an analysis of the Burden of Obstructive Lung Disease (BOLD) study, patients with a TB history had a 2.5-fold higher risk of airflow obstruction and a 2.1-fold higher risk of spirometric restriction [10]. A pooled analysis of 6 cohorts across 13 low- and middle-income countries (LMICs) found that individuals with a history of TB had lower lung function and a 4-fold increased risk of developing COPD compared to those without a TB history [14]. Furthermore, a meta-analysis revealed that a history of TB was associated with a 2.6-fold increased risk of future chronic airflow obstruction, with a pooled COPD prevalence of 21% among TB survivors [11].

Conversely, COPD itself has been identified as a risk factor for pulmonary TB, with a reported hazard ratio (HR) of 2.47 (95% confidence interval [CI], 2.21–2.76) [21]. Moreover, the use of inhaled corticosteroids (ICS) in COPD patients may further increase the risk of developing TB [22,23].

The dual burden of TB and COPD is particularly pronounced in LMICs, where TB incidence remains high and access to advanced respiratory care is limited [24]. In these settings, the long-term consequences of TB are more likely to manifest, contributing to persistent respiratory symptoms and airflow obstruction that accelerate COPD progression. This intersection not only complicates clinical management but also demands substantial healthcare resources [25]. Notably, the BOLD study demonstrated that the prevalence of airflow obstruction was 19.5% in Cape Town (TB prevalence: 15.4%), 15.2% in Nampicuan and Talugtug, Philippines (TB prevalence: 10.8%), and 7.0% in Pune, India (TB prevalence: 7.0%), underscoring the significant COPD burden in TB-endemic regions [10]. These epidemiological findings underscore the critical need for integrated public health strategies, including enhanced surveillance, improved diagnostic modalities, and targeted interventions, particularly in resource-limited regions [25]. Recognizing the epidemiological associations between TB and COPD is essential for reducing the global burden of chronic respiratory diseases.

## Pathogenesis of TB-COPD

### Molecular mechanisms

TB-COPD is driven by persistent immune activation that leads to progressive lung damage [26-28]. TB infection stimulates alveolar macrophage activation and neutrophil infiltration, which in turn release high levels of proinflammatory cytokines such as tumor necrosis factor-α, interleukin (IL)-1β, and IL-6 [26]. These cytokines promote granuloma formation and sustain inflammation even after TB treatment, ultimately causing structural lung damage and airway remodeling [27]. The chronic inflammatory response is further intensified by oxidative stress and excessive immune cell recruitment, contributing to long-term pulmonary dysfunction [29]. Notably, the inflammatory profile in TB-COPD differs from that observed in smoking-related COPD [30].

Matrix metalloproteinases (MMPs) play a pivotal role in the tissue destruction observed in TB-COPD [27-29,31]. MMP-1, MMP-8, and MMP-9 are highly expressed in TB-infected lungs, where they degrade extracellular matrix components, resulting in alveolar destruction and emphysema formation [29,31,32]. TB infection upregulates MMP expression in response to hypoxia and chronic inflammation, further exacerbating lung tissue damage. Studies have demonstrated that TB patients with elevated MMP levels exhibit more severe lung function impairment, along with increased fibrosis and remodeling [33]. Persistent dysregulation of MMPs even after TB treatment suggests a mechanistic link between post-TB disease and COPD [27,31].

Neutrophilic inflammation significantly contributes to airway remodeling and irreversible airflow obstruction in TB-COPD [26]. In TB patients, neutrophils are excessively recruited to the lungs, where they undergo necrotic cell death and release neutrophil extracellular traps (NETs), which consist of DNA, histones, and proteolytic enzymes [29,34,35]. Although NETs help contain bacteria, their excessive formation results in tissue necrosis, fibrosis, and airway remodeling. Additionally, oxidative stress and excessive neutrophil degranulation contribute to the progression of emphysema and persistent lung inflammation in TB-COPD [36,37].

### Structural lung damage, fibrosis, and airway remodeling

Structural lung damage and fibrosis in TB-COPD stem from chronic inflammation, excessive immune activation, and dysregulated tissue repair mechanisms following TB infection [26]. In severe cases, TB-COPD may present as extensive structural lung damage—often termed TB-destroyed lung—characterized by parenchymal destruction, traction bronchiectasis, and persistent airflow obstruction [38,39]. Granuloma formation—a hallmark of TB—leads to necrosis and cavitation, resulting in fibrotic scarring and architectural distortion of the lung parenchyma [40]. Fibrotic changes following TB infection are typically asymmetrical and heterogeneous, primarily affecting the upper lobes, which distinguishes TB-COPD from smoking-related COPD [41]. These fibrotic alterations cause traction bronchiectasis, pleural thickening, and permanent alveolar damage, contributing to restrictive lung function abnormalities and airflow obstruction [42].

Furthermore, post-TB scarring frequently results in bronchial stenosis and large airway obstruction, particularly in patients with a history of endobronchial TB [42]. Moreover, a previous study demonstrated that post-TB patients exhibit significantly lower maximal mid-expiratory flow, forced expiratory flow at 50% (FEF50), and FEF75, suggesting early small airway impairment [41].

Histological analysis of post-TB lungs revealed residual healed and fibrotic granulomas predominantly located along bronchovascular bundles [43]. These granulomas were encircled by fibrosis, resulting in bronchiolar narrowing, distortion, and occasional dilation. Additionally, adjacent pulmonary arterioles were partially incorporated into the fibrotic process, with multinucleated giant cells present, although no detectable organisms were found on special stains.

## Clinical manifestations

TB survivors frequently experience significant post-TB lung damage, which has been linked to poor outcomes such as accelerated lung function decline, persistent respiratory symptoms, and frequent healthcare visits within 1 year after completing treatment [44,45]. This persistent lung damage elevates the risk of developing TB-COPD, as structural and functional airway impairments can lead to chronic airflow obstruction and long-term respiratory complications.

**Fig. 1.**
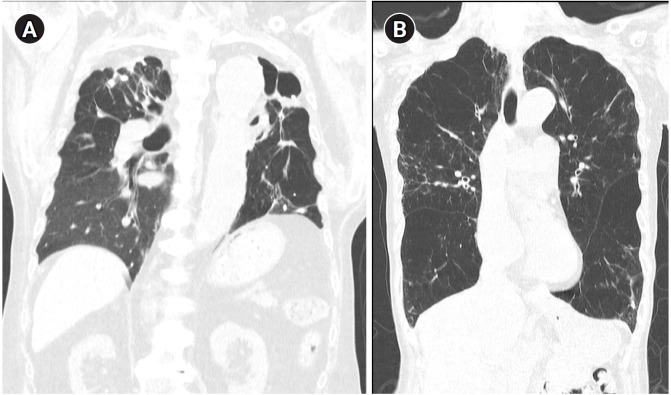
Representative computed tomography images illustrating typical radiologic features of tuberculosis-associated chronic obstructive pulmonary disease and smoking-related chronic obstructive pulmonary disease. (A) A patient with tuberculosis-associated chronic obstructive pulmonary disease shows fibrotic changes, cavitation, and traction bronchiectasis predominantly in the upper lobes. (B) A patient with smoking-related chronic obstructive pulmonary disease exhibits emphysematous lesions and airway wall thickening typical of smoking-related chronic obstructive pulmonary disease.

TB-COPD exhibits clinical manifestations distinct from those of COPD caused by tobacco smoking. TB-COPD primarily affects younger individuals following TB infection, presenting with fixed airflow obstruction, cavitation, and fibrosis (Fig. 1A). In contrast, non-TB-COPD predominantly affects older adults with a history of smoking or environmental exposure and is characterized by fixed or partially reversible airflow obstruction, airway wall thickening, and emphysema (Fig. 1B) [22]. Hemoptysis may occur in TB-COPD patients, particularly in those with secondary infections, bronchiectasis, or chronic pulmonary aspergillosis [22]. TB-COPD may be static or progressive with recurrent exacerbations, whereas non-TB-COPD typically follows a progressive course with recurrent acute exacerbations [22].

Studies indicate that TB-COPD may lead to poorer outcomes compared to smoking-related COPD. An analysis of the Korean National Health Insurance Service database (2010–2017) revealed that TB survivors had a significantly higher risk of developing COPD (adjusted HR [aHR], 1.63; 95% CI, 1.54–1.73) and of COPD-related hospitalization (aHR, 2.03; 95% CI, 1.81–2.27) [46]. Furthermore, TB-COPD patients were more likely to require COPD-related hospitalization compared to non-TB COPD patients (aHR, 1.84; 95% CI, 1.17–2.92). Additionally, an analysis of a multicenter COPD cohort in South Korea found that COPD patients with a history of TB experienced worse outcomes, including more severe symptoms (COPD assessment test: 16.1 vs. 14.8, P=0.002), poorer quality of life (St. George’s Respiratory Questionnaire for COPD: 36.6 vs. 32.6, P<0.001), and a higher prevalence of exacerbations (28.8% vs. 23.5%, P=0.031) as well as severe exacerbations requiring hospitalization (3.9% vs. 1.5%, P=0.002) [47]. Moreover, a single-center study conducted in China reported that TB-COPD patients were more likely to have bronchiectasis and emphysema and experienced more significant breathlessness and frequent exacerbations compared to non-TB-COPD patients [48]. Elevated IL-6 levels and the presence of bronchiectasis have been identified as risk factors for future exacerbations in TB-COPD [39,49]. Further investigations involving larger populations and longer follow-up periods are needed to enhance the generalizability of these findings.

## Management strategies

The treatment of TB-COPD is primarily guided by current COPD management guidelines [22]. Long-acting bronchodilators constitute the cornerstone of therapy, and ICS should be reserved for patients with high blood eosinophil levels and frequent exacerbations [3]. However, the use of ICS may contribute to TB relapse in COPD patients with radiologic sequelae of prior TB [50]. In patients with central airway obstruction, inhaled medications may have limited efficacy; in such cases, interventional bronchoscopic procedures—including bronchoscopic dilation, airway stenting, argon plasma coagulation, and electrocautery—can be beneficial [22]. For patients with recurrent hemoptysis due to bronchiectasis, bronchial artery embolization or surgical resection of the affected area may be necessary [22].

Few studies have addressed the treatment of TB-COPD. The indacaterol effectiveness in COPD patients with tuberculosis history (INFINITY) study, a randomized double-blind placebo-controlled trial, evaluated the efficacy and safety of indacaterol in patients with TB-COPD [51]. After 8 weeks, indacaterol significantly improved forced expiratory volume in 1 second (+140 mL, P<0.001), dyspnea scores, and health status compared to placebo, while maintaining a comparable safety profile.

Notably, a large proportion of patients enrolled in this study were never-smokers, which enhances the relevance of the findings. These results suggest that indacaterol is a beneficial treatment option for TB-COPD. A post-hoc analysis of the INFINITY study identified factors associated with improved lung function in response to indacaterol, revealing that a shorter smoking history and a high bronchodilator response were linked to better outcomes [52]. Moreover, the use of long-acting muscarinic antagonists (LAMAs) has also shown benefits in TB-COPD patients. A retrospective, single-center study analyzing the mortality benefits of LAMA therapy in TB-COPD found that the LAMA group had a significantly lower 5-year mortality rate compared to the non-LAMA group (3.1% vs. 14.1%, P=0.039) [53]. Additionally, an analysis of the Korean National Health Insurance claims database found that tiotropium use was associated with reduced mortality in patients with TB-destroyed lungs, although the study included both COPD and non-COPD patients (HR, 0.560; 95% CI, 0.38–0.82) [54].

## Conclusion

TB-COPD is a distinct clinical entity characterized by unique structural and inflammatory features compared to smoking-related COPD. Numerous studies have demonstrated a heightened likelihood of chronic lung impairment following TB infection, resulting in persistent airflow limitation and increased healthcare needs. The underlying mechanisms include sustained immune activity, elevated production of MMPs, and pronounced neutrophil-driven inflammation, ultimately leading to fibrotic scarring and airway remodeling. Clinically, individuals with TB-COPD often present at a younger age, exhibit marked fibrotic changes in the lung, and may experience hemoptysis due to coexisting bronchiectasis. Management generally follows standard COPD recommendations, emphasizing long-acting bronchodilators and cautious use of inhaled corticosteroids based on individual risk profiles. Both indacaterol and tiotropium have demonstrated benefits in improving pulmonary function, reducing exacerbations, and lowering mortality in these patients. In certain cases, interventional or surgical procedures may be necessary to address severe airway obstructions or persistent hemoptysis.

In addition, early TB detection, prompt and effective TB treatment (including latent TB management), and the development of host-directed therapies can help limit the progression of post-TB lung disease [55]. By combining these approaches, clinicians may improve patient outcomes, reduce the global burden of TB-COPD, and enhance the quality of life for affected individuals.
